# Macular vascular changes in pregnant women with gestational diabetes mellitus by optical coherence tomography angiography

**DOI:** 10.1186/s12886-021-01927-1

**Published:** 2021-04-09

**Authors:** Guodong Liu, Fang Wang

**Affiliations:** Department of Ophthalmology, Shanghai Tenth People’s Hospital affiliated with Tongji University, 301 Middle Yan Chang Road, Shanghai, 200072 People’s Republic of China

**Keywords:** Gestational diabetes mellitus, Optical coherence tomography angiography, Retinal capillary, Vascular density

## Abstract

**Background:**

Retinal capillary is vulnerable to diabetes, whether gestational diabetes mellitus (GDM) eyes without clinical retinopathy have capillary abnormalities has not been well studied. To observe the microvasculature changes in eyes of GDM women compared with normoglycemic pregnant women and non-pregnant women by optical coherence tomography-angiography (OCT-A).

**Methods:**

GDM women, age-matched normoglycemic pregnant women and non-pregnant women were included in this study. All subjects were examined by OCT-A, vascular density and macular foveal parameters were measured automatically.

**Results:**

Thirty eight non-pregnant women (NC group), thirty pregnant women without GDM (PC group), and thirty one GDM women (GDM group) were included in this study. There was a significant reduction of vascular density in superficial capillary layer, but an increase in deep capillary layer in PC and GDM groups (*P* < 0.001). When in terms of the average vascular density, the difference was insignificant among these three groups. Although all the measurements were similar between PC and GDM groups, more capillary “dropout” changes were detected in GDM group. Unexpectedly, the abnormal changes of central macular thickness thinning and foveal avascular zone enlargement seen during pregnancy were improved when compared to PC group.

**Conclusions:**

The changes of vascular density implied the redistribution of capillary network from superficial to deep layer under pregnancy and GDM states. Although the transient hyperglycemia aggravates the changes of capillary “dropout”, GDM group revealed the improvement of central macular thickness thinning and foveal avascular zone enlargement during pregnancy.

## Background

Gestational diabetes mellitus (GDM) is defined as variable hyperglycemia during the second and third trimester of pregnancy [1]. The disorder can lead to serious burdens for both adults and infants, including pre-eclampsia, Type 2 diabetes, cardiovascular diseases and neonatal hypoglycemia [2–5]. GDM also causes abnormal changes on eyes such as eyelid chloasma, increased cornea thickness, refractive change, diabetic retinopathy, and papilledema [6, 7]. Among the ocular abnormalities, retinal changes are the most commonly detected as the microvascular changes were closely related to GDM severity and diabetes courses [6, 8]. Previous studies reported vascular changes in small vessels, displaying retinal vessels tortuosity [9], narrower retinal vessel caliber, larger retinal vessel branching angle and reduced vasodilatory response in GDM eyes [8, 10, 11]. However, most of GDM eyes didn’t show any clinical retinopathy changes, thus whether those GMD eyes have similar capillary abnormalities has not been well studied because of the lack of imaging method.

Optical coherence tomography-angiography (OCT-A) is the emerging technology to image vascular changes within different vascular layers in vivo [12]. OCT-A has shown great advantages in the measurement of capillary density [13, 14] and visualization of non-perfusion area, vascular morphology changes and neovascularization [15, 16]. Recently, studies reported vascular density changes in normal pregnant eyes [17–19], but there were no similar studies in GDM eyes.

Furthermore, previous studies found microvascular abnormalities in diabetes eyes without clinical retinopathy by OCT-A, such as the microaneurysms [20–22]. Thus, we wonder whether GDM eyes without clinical retinopathy had similar ocular microvascular changes with the use of OCT-A. Therefore, the present study aims to observe the retinal microvasculature changes in eyes of GDM, when compared to normoglycemic pregnant women and non-pregnant women by OCT-A.

## Methods

### Subjects and groups

From September 2019 to April 2020, a total of 99 individuals were enrolled in this study. The subjects were separated into three groups: 38 non-pregnant women as normal controls (NC); 30 pregnant females without diabetes as pregnant controls (PC); and 31 women with GDM as GDM group. None of the subjects showed any ophthalmic (including intraocular pressure higher than 20 mmHg, or any medium opacity) or systemic diseases, except for GDM in the GDM group. Exclusion criteria also included diabetes history prior to gestation, high blood pressure (140/90 mmHg), or albuminuria in pregnant women, best-corrected visual acuity (BCVA) less than 20/20 or ametropic higher than 3 diopters.

### GDM definition

GDM was diagnosed by the oral glucose tolerance test according to World Health Organization criteria: using a 75 g oral glucose tolerance test after overnight fasting (8 to 10 h) at 26–28 weeks gestation. While fasting glucose was ≥7.0 mmol/L and/or 2-h post-glucose was ≥7.8 mmol/L, GDM was diagnosed [23, 24].

### Ocular examinations

All subjects underwent a complete ophthalmic examination including BCVA, slit lamp biomicroscopy, intraocular pressure, fundus photography and OCT-A. We used a 45° non-mydriatic retinal camera (Canon CR-1, Canon Inc., Japan) to get digital fundus photographs in a darkened room without pharmacologic dilation.

OCT-A (Optovue Inc. Fremont, CA) with a light source spectrum of 840 nm, a bandwidth of 45 nm and axial resolution of 5 um was used in the study. The module of “Angio Retina” centered on the fovea within a 3 × 3 mm area was chosen to image macular microvasculature through horizontal and vertical scans in both eyes. The software of algorithm of split-spectrum amplitude-decorrelation angiography (SSADA) was used to segment the capillary system into two layers automatically: superficial retinal capillary plexus and deep retinal capillary plexus.

The superficial retinal capillary plexus starts from inner limiting membrane to 9 mm beneath the inner plexiform layer, and deep retinal capillary plexus starts from inner plexiform layer with an offset of 9 um to the outer boundary at 9 mm beneath the outer plexiform layer. High imaging quality was obtained through using “Follow-up” and “Tracking-on” modes during OCT examination process. Motion correction technology was used to remove motion artifacts through horizontal and vertical scans. Images with quality signal strength index [SSI] higher than 80 were included in the following data analysis.

### Measurements of vascular density

Vascular density was defined as the proportion of the examined area occupied by retinal vessels. Fovea within 1.0 mm ring, parafovea between 1.0 mm and 3.0 mm, and whole-image area within 3.0 mm around the fovea were evaluated, respectively. The vascular density in superficial and deep layers was automatically quantified using flow density map software of AngioAnalytics (version 2017.1.0.155).

### Measurements of the macular fovea area

Foveal avascular zone (FAZ) was defined by the borders of the vascular area, and the area size was measured automatically using “nonflow area” pattern. Central macular thickness (CMT) was the average thickness within central 1 mm area from inner limiting membrane to retinal pigment epithelium layer. Acircularity index (AI) was the index to evaluate macular circular regularity; foveal density (FD) was defined as the vascular density within a 300 μm width ring surrounding the FAZ.

### Statistical analysis

The statistical analysis was performed with the Statistical Package for the Social Sciences (SPSS) for Windows (version 27.0; IBM Inc.). Differences of vascular density, FAZ area, CMT, AI and FD among three groups were tested by One-way ANOVA, with a post hoc comparison performed using the Dunnett’s test. Values were presented as the mean ± standard deviation, the criterion significance was assessed at *P* < 0.05 level.

## Results

### Demographics

Among the 99 Chinese women, there were 38 non-pregnant women (NC group), 30 pregnant women without GDM (PC group), and 31 pregnant women with GDM (GDM group) were enrolled in this study. Among them, 14 eyes in NC group, 2 eyes in PC group and 3 eyes in GDM group were excluded because of the poor imaging quality mainly due to the motion artifacts and lower ISS less than 80. Thus, a total of 62 eyes in NC group, 58 eyes in PC group, and 59 eyes in GDM group were included for analysis in this study. The difference of mean age was insignificant among the three groups, which was 30.6 ± 4.9 years (range, 24–41 years) in NC group, 30.7 ± 4.9 years (range, 20–43 years) in PC group, and 30.6 ± 2.8 years (range, 25–41 years) in GDM group. The mean pregnancy duration was 34.9 ± 6.4 weeks in PC group, and 33.2 ± 6.2 weeks in GDM group, respectively (*P* = 0.199). None of the GDM group had the history of pre-pregnancy diabetes. The average GDM duration was 96.6 ± 28.3 d and the GMD women were able to control blood sugar well through diet or insulin therapy. Among the GDM group, four GMD women were treated with insulin injections, and the others were under diet control.

### Measurements of vascular density

**Fig. 1:**
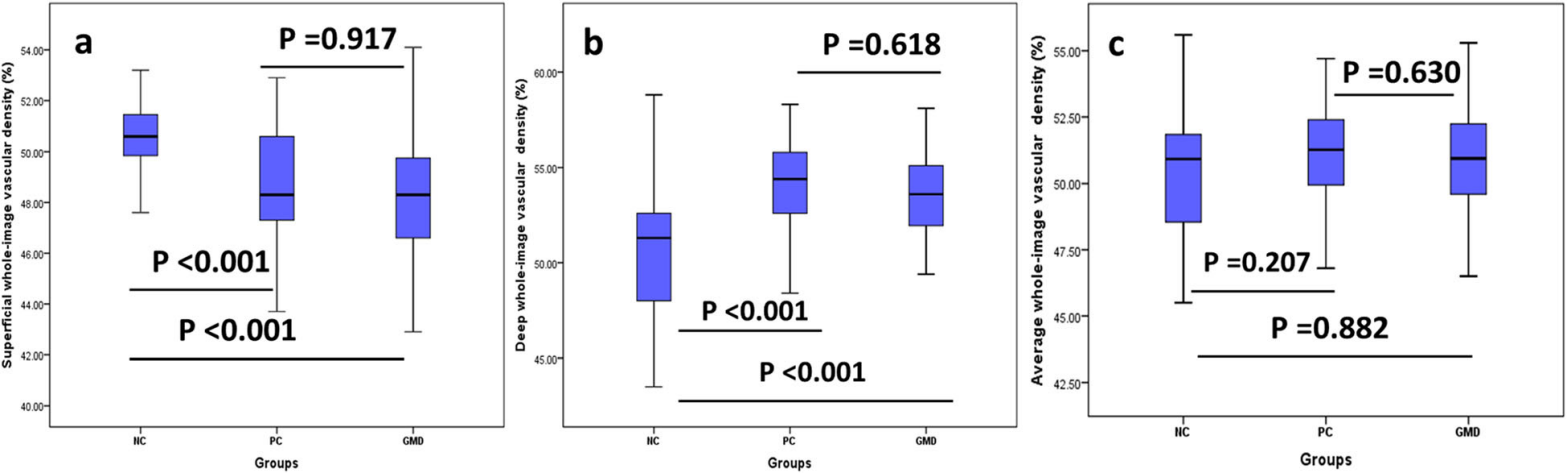
Measurements of whole-image vascular density among non-pregnant women (NC group), pregnant women without GDM (PC group), and pregnant women with GDM. The measurement of superficial vascular density in PC and GDM groups was lower than NC group (*P* < 0.001, (**a**)), but the vascular density in deep layer was significantly higher than NC group (*P* < 0.001, (**b**)). The average vascular density was calculated by the measurements of vascular density in superficial and deep layers. There was no significant difference of average vascular density among the three groups (*P* > 0.05, (**c**)). And all the values were similar between PC and GDM groups (*P* > 0.05)

The whole-image vascular density in superficial retinal capillary network was significant difference among the three groups (*P* < 0.001, Table 1). While the superficial vascular density in NC group was higher than both PC and GDM group (*P* < 0.001, Fig. 1a), there was no significant difference between PC and GDM group (*P* = 0.917, Fig. 1a).

**Table 1:** Values of vascular density measurement

Density(%)	NC	PC	GDM	P
Whole image (S)	50.4 ± 1.5	48.5 ± 2.4	48.2 ± 2.6	< 0.001
Whole image (D)	50.6 ± 3.5	53.9 ± 2.6	53.3 ± 3.1	< 0.001
Average Whole	50.5 ± 2.2	51.2 ± 1.9	50.7 ± 2.3	=0.246
Fovea (S)	24.0 ± 6.3	14.5 ± 4.3	16.5 ± 6.1	< 0.001
Fovea (D)	32.2 ± 6.9	27.9 ± 6.8	30.3 ± 7.5	=0.004
ParaFovea (S)	53.2 ± 1.6	51.8 ± 2.8	51.5 ± 2.6	< 0.001
ParaFovea (D)	53.0 ± 3.6	57.1 ± 2.7	56.2 ± 2.7	< 0.001

*S* superficial capillary layer, *D* deep capillary layer

The whole-image vascular density both in PC and GDM groups was much higher than NC group (*P* < 0.001, Fig. 1b), but without significant difference between PC and GDM groups (*P* = 0.618, Fig. 1b). In terms of the average vascular density, there was no significant difference among the three groups (*P* = 0.246, Fig. 1c), which was 50.5 ± 2.2% in NC group, 51.2 ± 1.9% in PC group, and 50.7 ± 2.3% in GDM group, respectively.

**Fig. 2:**
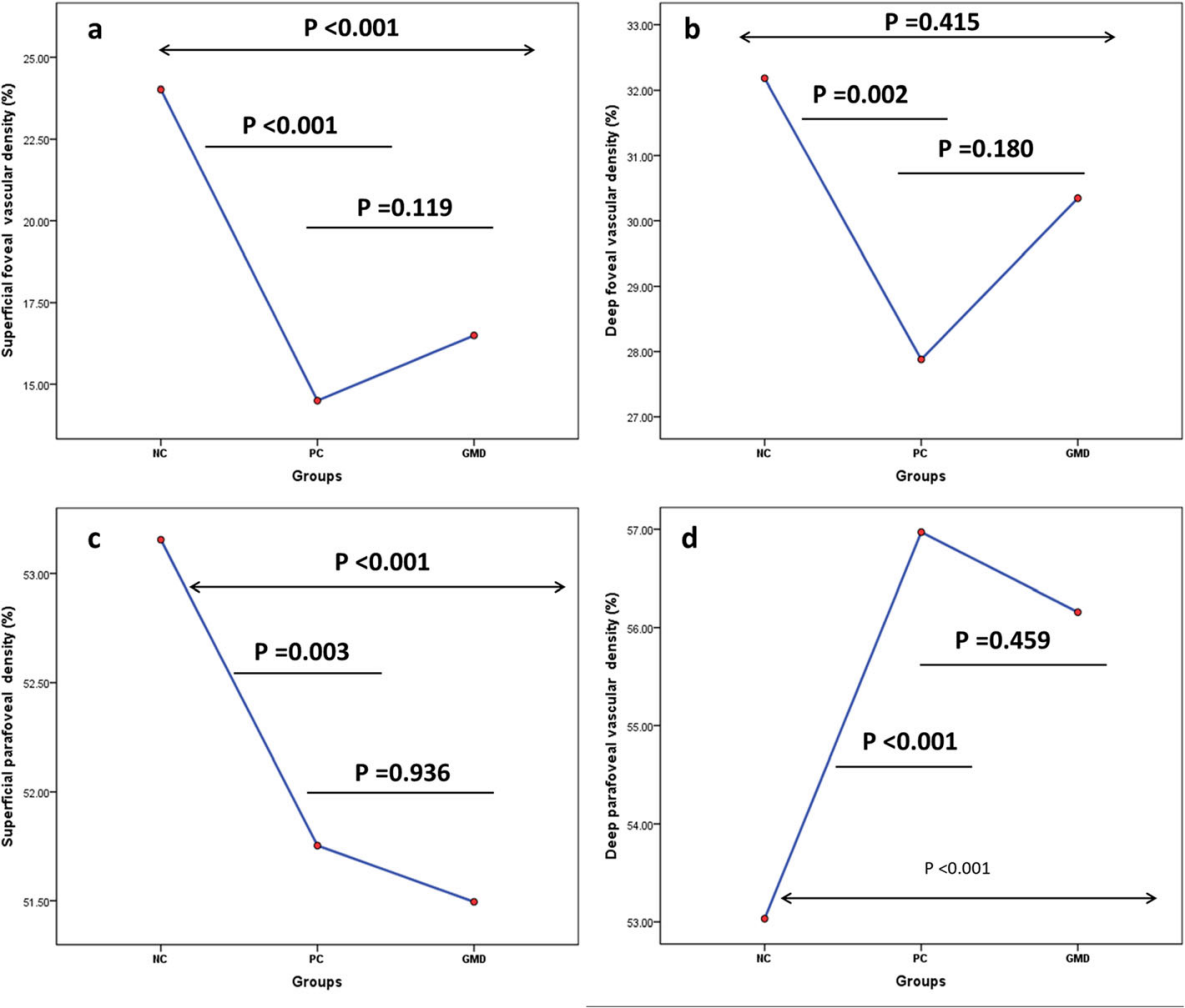
Measurements of foveal and parafoveal vascular density in superficial and deep layers among NC, PC and GDM groups. The foveal vascular density in PC and GDM groups was lower than NC group both in superficial and deep layers (**a**, **b**). While the parafoveal vascular density in PC and GDM groups was lower than NC group in superficial layer (**c**), the measurement was higher than NC group in deep capillary layer (**d**). All the measurements were similar between PC and GDM groups (*P* > 0.05)

In accordance with whole-image vascular density in superficial layer, foveal and parafoveal vascular density in NC group was much higher than that in PC (*P* < 0.001 for both, Fig. 2a) and GDM groups (*P* < 0.01 for both, Fig. 2c). And the difference of foveal and parafoveal vascular density in PC and GDM groups was still insignificant (*P* = 0.119, Fig. 2a; *P* = 0.180, Fig. 2c; respectively).

The foveal vascular density in deep layer was higher in NC group than that in PC group (*P* = 0.002, Fig. 2b), but without significant difference when compared to GDM group (*P* = 0.415, Fig. 2b). (However, the parafoveal vascular density in PC and GDM groups was much higher than that in NC group (*P* < 0.001 for both, Fig. 2d). The difference of both foveal and parafoveal vascular density in deep layer was insignificant between PC and GDM groups (*P* = 0.18, Fig. 2b; *P* = 0.459, Fig. 2d).

### Measurements of FAZ and CRT

**Fig. 3:**
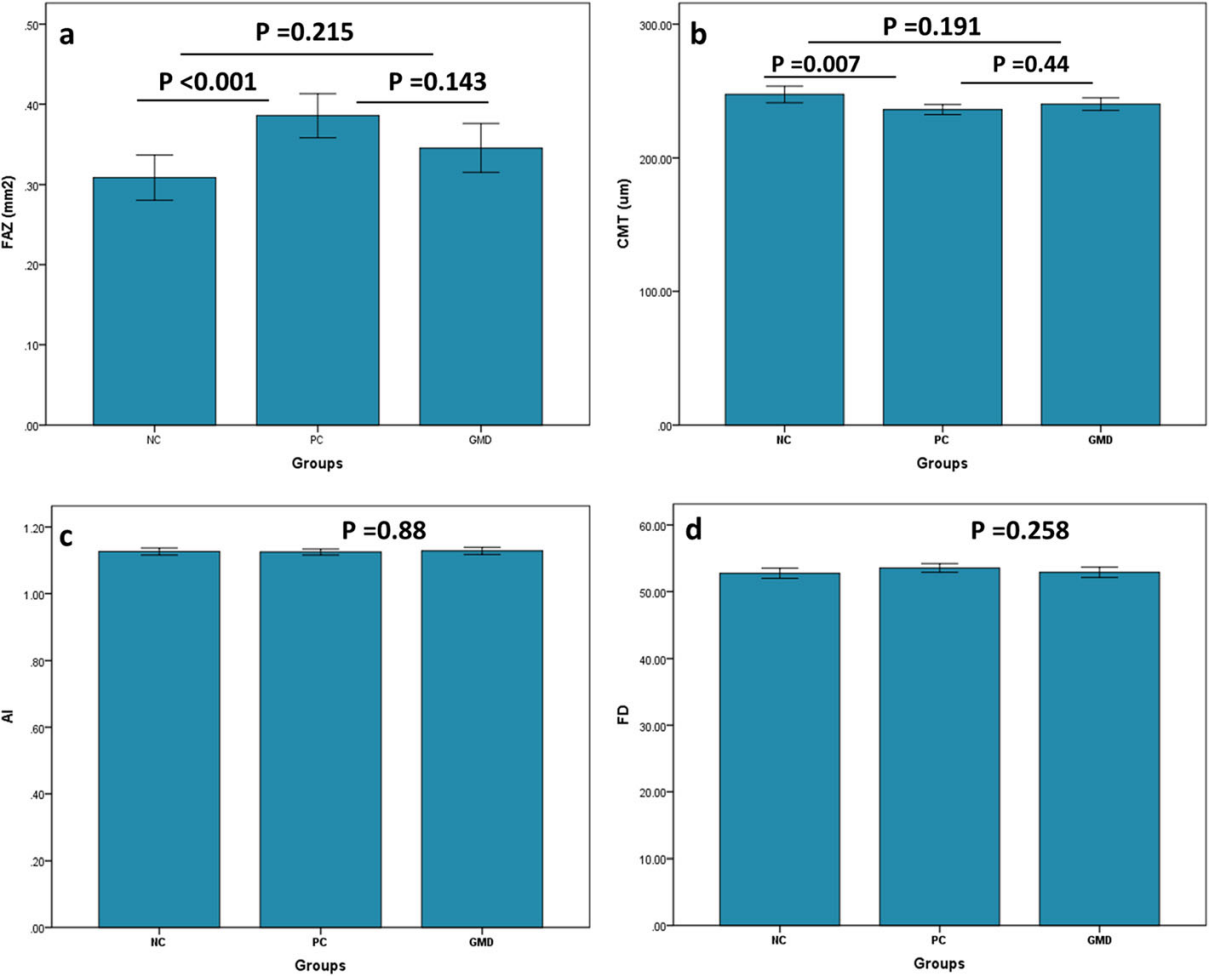
Measurements of foveal avascular zone (FAZ), central macular thickness (CMT), acircularity index (AI) and foveal density (FD) among NC, PC and GDM groups. PC group showed larger FAZ size and thinner CMT, but the difference between NC and GDM groups was insignificant (**a**, **b**). There was no significant difference of the measurements of AI and FD among three groups (**c**, **d**)

In PC and GDM groups, there were no significant vascular abnormality found within macular circular area, but the FAZ size in PC group was larger than NC group (*P* < 0.001, Fig. 3a). Unexpected, hyperglycemia state mitigated the difference with insignificant difference between GDM group and PC group (*P* = 0.125, Fig. 3a). Conversely, the PC group showed smaller CRT when compared to NC group (*P* = 0.007, Fig. 3b). But the differences between GDM groupand NC group or PC group were insignificant (*P* = 0.191, *P* = 0.44, respectively; Fig. 3b). The detailed values of FAZ and CMT are listed in Table 2.

**Table 2:** Comparisons of FAZ, CRT, AI and FD among three groups

Parameters	NC	PC	GDM	P
FAZ (mm^2^)	0.31 ± 0.11	0.39 ± 0.10	0.35 ± 0.12	= 0.001
CMT (um)	247.3 ± 24.5	235.9 ± 14.3	240.1 ± 18.1	= 0.007
AI	1.13 ± 0.04	1.12 ± 0.03	1.13 ± 0.04	= 0.880
FD (%)	52.7 ± 2.9	53.5 ± 2.5	52.9 ± 2.9	= 0.258

*FAZ* Foveal avascular zone, *CMT* Central macular thickness, *AI* acircularity index, *FD* foveal area density within 300 μm width ring, *NC* non-pregnant women, *PC* pregnant women without GDM, *GDM* Gestational diabetes mellitus

### Measurements of AI and FD

In addition to FAZ and CMT, AI was the index to evaluate macular circular irregularity, and FD was used to assess the foveal density within 300 μm width ring surrounding the FAZ. Unlike FAZ and CMT, the difference of AI and FD was insignificant among the three groups (*P* = 0.880 for AI, Fig. 3c; *P* = 0.258 for FD, Fig. 3d).

Apart from the difference of vascular density in superficial and deep layers, we detected capillary “dropout” changes exclusively in superficial capillary layer, but without any other pathological microvascular changes, such as microaneurysm, hemorrhagic spot or exudate.

### Examples of eyes from NC, PC and GMD groups

**Fig. 4:**
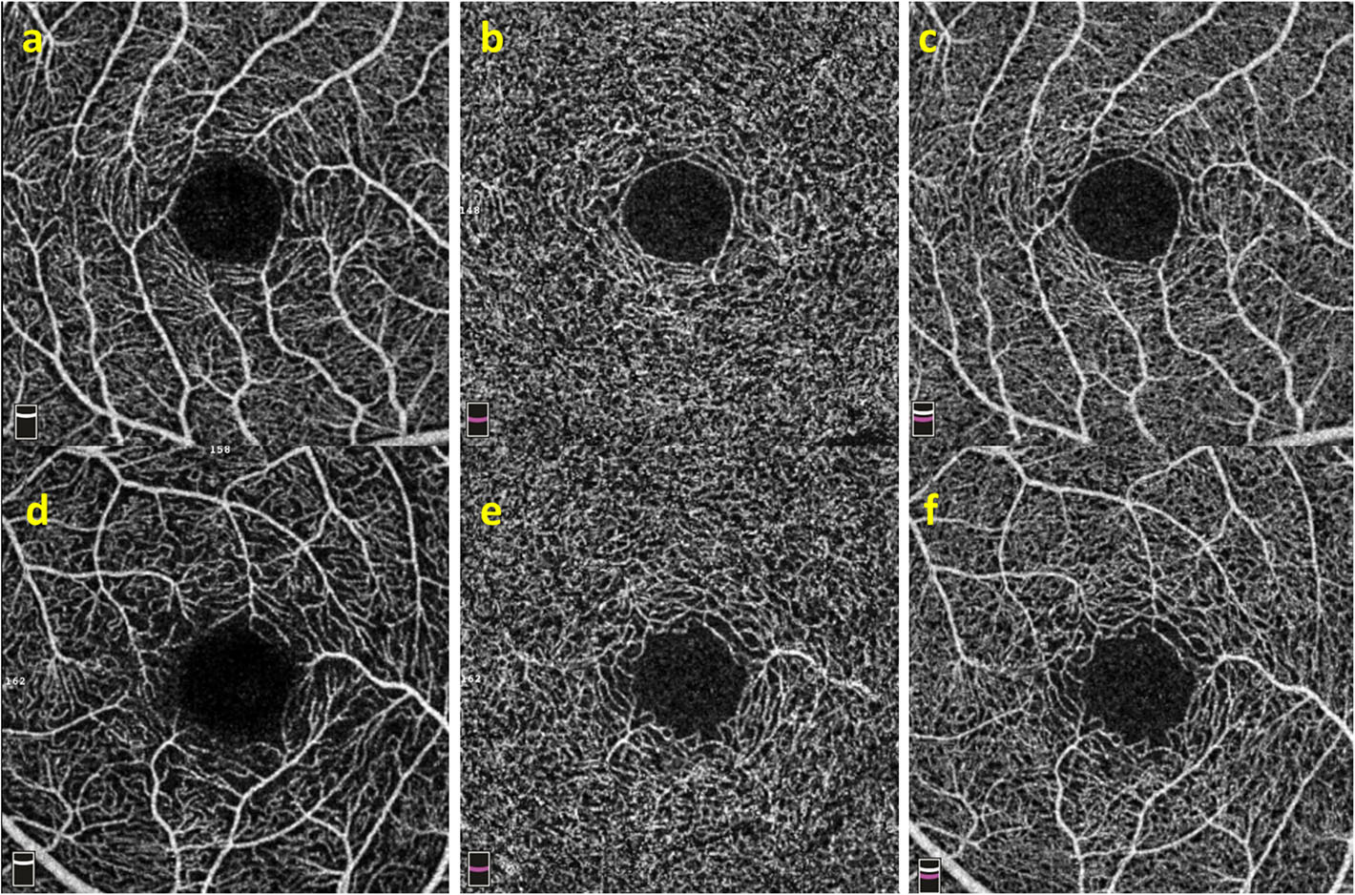
Retina capillary in eyes of NC and PC groups. **a**-**c** An example of eye with regular capillary morphology both in superficial, deep and full capillary layers from NC group. **d**-**f** An example with sparse capillary network in superficial layer, denser vascular branches in deep layer, and irregular macular circular ring in full capillary layer from PC group

**Fig. 5:**
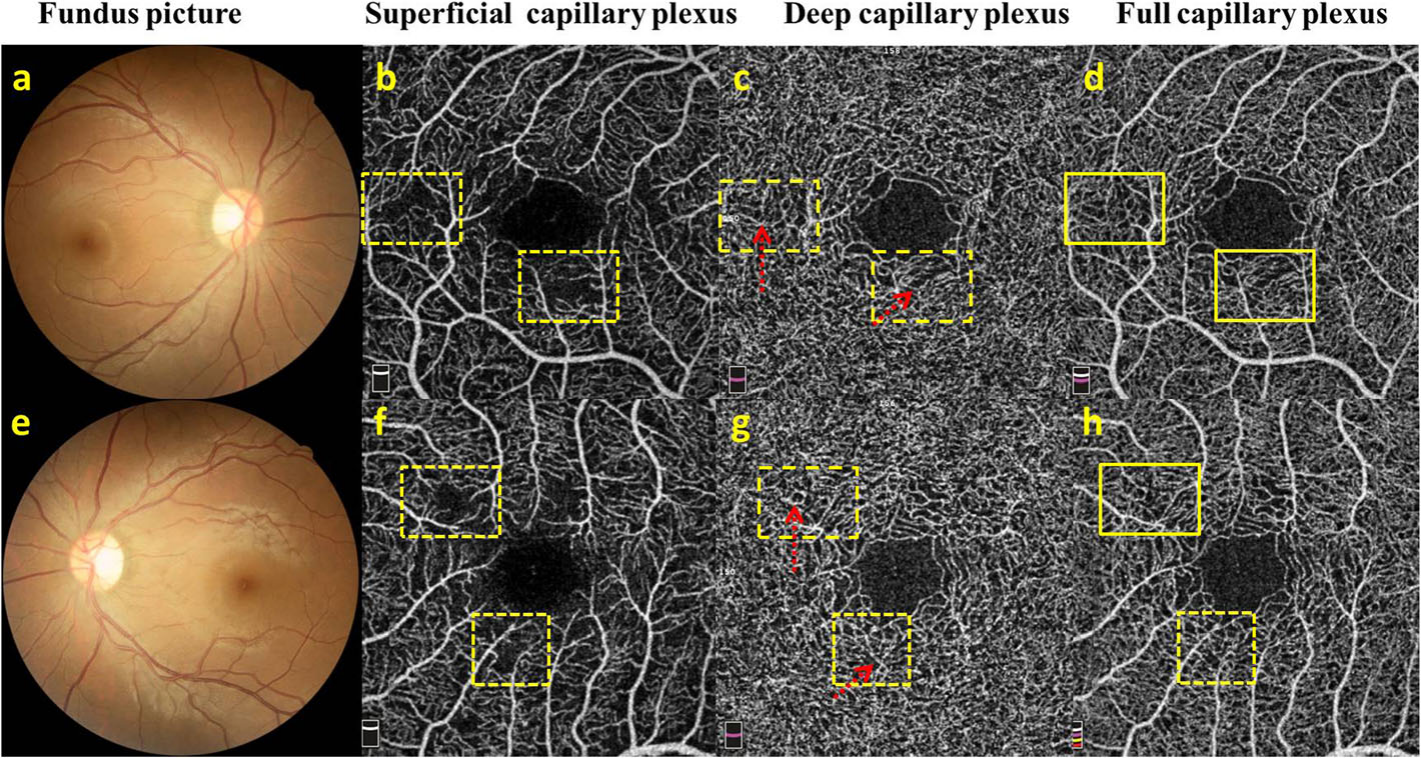
Retina capillary from a GMD female. **a**, **e** Fundus pictures show tortuous retinal arterioles without any microaneurysms, intraretinal hemorrhages, cotton wool spots or hard exudated abnormalities. **b**, **f** Capillary “dropout” was shown in superficial layer (yellow dotted line box). **c**, **g** The capillary branches within the corresponding area originating from superficial layer could be detected in deep capillary layer (red dotted arrows). **d**, **h** Relatively normal capillary network (yellow solid line box) was found in full capillary plexus image

An example of NC eye with regular capillary morphology both in superficial and deep layers was depicted in Fig. 4a-c. An example of PC eye showed relatively loosened vascular branches in superficial layer, but dense vascular branches in deep layer, and irregular macular circular ring (Fig. 4d-f). There were no clinical changes on the fundus of the two eyes from a GMD female (Fig. 5a, e), but we detected capillary “dropout” in superficial layer (Fig. 5b, f) by OCT-A, and vertical branches in deep layer originating from superficial layer (Fig. 5c, g). But the full capillary plexus were relatively normal (Fig. 5d, h).

## Discussion

The current study aimed to evaluate the macular microvascular changes in GDM eyes compared with normoglycemic pregnant women and non-pregnant women by OCT-A. Previously, clinical studies revealed microaneurysms in diabetic eyes without clinical retinopathy on OCT-A images [20–22]. Although most GDM individuals can recover from high blood glucose levels after delivery, whether this transient hyperglycemia state causes microvascular abnormalities were little known until now.

Our results detected a significant decrease of vascular density and capillary dropout in superficial vascular layer both in PC and GDM groups, but without any other microvascular changes related to diabetic retinopathy in GDM eyes, such as microaneurysm, hemorrhagic spot or exudate changes on both OCT-A and fundus photographs.

Two reasons accounting for the lack of microaneurysm in GDM eyes was the short duration of diabetes and well controlled blood glucose. Most of the GDM females were able to control blood sugar well through diet control or insulin therapy. As a result, the transient GDM duration and mild hyperglycemia state could not cause obvious capillary abnormalities. Another possible explanation was the disadvantage of OCT-A to detect microaneurysm that the algorithm has blood flow requirement to discern the changes [25].

Although we detected similar vascular measurements in superficial capillary layer of PC and GDM groups, the capillary “dropout” was more common in GDM group. With respect to the vascular density in deep layer, we detected opposite results. Both PC and GDM groups had higher vascular density than that in NC group. More specifically, the increase was found in the parafoveal area, but with a lower vascular density in the foveal area. Similarly, all the difference of deep measurements was insignificant between PC and GDM groups.

Previously, the changes of vascular density in pregnant women have been reported, that whole macula vascular density both in superficial and deep layers were significantly higher in Kiziltunc’s study [19], or lower superficial vascular density and higher deep vascular density in healthy pregnant women by OCT-A in Chanwimol’s study [18]. The reasons accounting for the variable results were different inspection equipment and variable inclusion criteria, such as imaging quality. One of the studies used images with SSI higher than 30 [19], which was not accurate enough for density measurement. Image artifacts are the common index to cause incorrect interpretations and lead to inaccurate measurement of vascular density [26, 27]. But our study only used pictures with a SSI higher than 80 to analyze measurements to guarantee the quality of pictures.

Likewise, there were studies that explained the changes of vascular density in pregnant women, mainly on the increase of blood volume and elevated hydrostatic retinal pressure during pregnancy [18, 28, 29]. The high hydrostatic pressure could cause the constriction of retinal vessels resulting in a decrease of vascular density [18, 30]. However, the similar mechanism cannot explain the paradoxical results of increased vascular density in deep capillary layer. We assumed the mechanism of blood flow autoregulation to explain the opposite results through blood flow redistribution.

The autoregulation theory has been widely reported in previous studies: retinal blood flow could remain relatively stable under the changes of blood pressure, physical exercise or body position changes [31–33]. When it comes to the results in this study, the insignificant changes of average whole-image vascular density supports the theory of blood flow autoregulation that whole macular blood flow kept relatively stable, regardless of the decreased vascular density in superficial capillary layer and increase in deep capillary layer.

The related studies reported the inconsistent changes of vascular density within different capillary layers under elevated intraocular pressure [34, 35], and found more pronounced vasodilation in the deep capillary layer when compared to the superficial layer [34, 36]. Significant vasodilation has been found in deep capillary layer in response to the elevated intraocular pressures [37, 38] and flicks stimulus [34, 36]. So the concept of capillary regulation has been proposed that retinal capillary without smooth muscle also has the capacity to regulate blood flow positively. The mechanism mainly relies on the pericytes and astrocytes that induce capillary regulation through cell constriction [39–41].

The higher vasodilation in the deep capillary layer was consistent with the results in our present study that lower vascular density in superficial layer but higher vascular density in deep layer in PC and GDM groups. In addition, excess sex hormones could decrease intraocular pressure during pregnancy [7, 42], thus higher ocular perfusion pressure was caused by elevated hydrostatic pressure and lower intraocular pressure. Then the theory of blood flow redistribution can well explain the results that the average vascular density in PC and GDM groups were unchanged, but the vascular density in superficial layer was decreased with the increase of vascular density in deep layer under the circumstance of elevated ocular perfusion pressure.

Although we detected a decrease of the foveal capillary density both in PC and GDM groups, the changes of FD were insignificant among the three groups. The variable results were mainly due to different measured areas that foveal region refers to the area within 1.0 mm ring, while FD region refers to the area within the 300 μm width ring surrounding the FAZ. As the greater FAZ size has been found in PC and GDM groups, the proportion within foveal region occupied by retinal vessels was less.

Although we detected irregular macular ring in PC and GDM groups, the difference of AI was insignificant among these three groups. But the PC and GDM groups showed larger FAZ size and thinner CMT when compared to the NC group. The negative correlation between FAZ and CMT has been investigated in our previous study [43], so a larger FAZ size combined with thinner CMT was expected. The enlargement of FAZ size can possibly be attributed to the decrease of foveal vascular density both in superficial and deep capillary layers. Unexpectedly, the expanded FAZ and thinned CMT were mitigated under hyperglycemia state that similar FAZ and CMT were acquired in GDM group when compared to the NC and PC groups.

In addition to the decrease of vascular density, the capillary “dropout” area has been exclusively detected in superficial layer both in PC and GDM groups. But the capillary “dropout” was different from the avascular area shown in diabetic retinopathy, which was the capillary occlusion both in superficial and deep capillary layers [44, 45]. While the capillary “dropout” in PC and GDM groups was supposed to be the vascular remodeling from superficial capillary layer to deep capillary layer, as the relatively regular capillary branches were found on full capillary plexus layer. Regardless of the similar changes of superficial vascular density between PC and GDM groups, more capillary “dropout” was detected in GDM groups.

Therefore, pregnant state changes the distributed patterns of capillary layers that the vascular densities of superficial and deep layers were similar in NC group, but the vascular density in deep vascular layer was much higher than that in superficial layer in both PC and GDM groups. Although no clinically pathological changes related to diabetic retinopathy were found in GDM group, the transient hyperglycemia lead to more capillary “dropout” changes in superficial layer.

There were limitations worth noting in the current study. In spite of the fact that most of the retinal abnormalities can recover after delivery of a baby, changes of vascular density were not followed up after pregnancy. Although it is well known that increasing estrogen and progesterone were the factors causing vasoconstriction [46, 47], we didn’t measure hormone levels among the three groups.

## Conclusion

The current study provides the evidence that the pregnancy state profoundly impacts the distributions of capillary branches from superficial capillary layer to deep capillary layers. The redistribution caused capillary “dropout” changes in superficial capillary layer which was aggravated by transient hyperglycemia state. These findings contribute to the understandings of retinal capillary auto-regulated mechanism that facilitates retina to cope with hemodynamic changes under pregnancy or GMD states.

## Data Availability

The datasets used and/or analyzed during the current study available from the corresponding author on reasonable request.

## Abbreviations

GDM: Gestational diabetes mellitus
NC: Normal controls
PC: Pregnant controls
OCT-A: Optical coherence tomography-angiography
BCVA: Best corrected visual acuity
FAZ: Foveal avascular zone
CMT: Central macular thickness
AI: Acircularity index
FD: Foveal density

## References

[1] American Diabetes A (2020). 2. Classification and diagnosis of diabetes: standards of medical care in diabetes-2020. Diabetes Care.

[2] Zhu Y, Zhang C (2016). Prevalence of gestational diabetes and risk of progression to type 2 diabetes: a global perspective. Curr Diab Rep.

[3] Metzger BE, Coustan DR, Trimble ER (2019). Hyperglycemia and adverse pregnancy outcomes. Clin Chem.

[4] Kampmann U, Madsen LR, Skajaa GO, Iversen DS, Moeller N, Ovesen P (2015). Gestational diabetes: a clinical update. World J Diabetes.

[5] Ferranti EP, Jones EJ, Hernandez TL (2016). Pregnancy reveals evolving risk for cardiometabolic disease in women. J Obstet Gynecol Neonatal Nurs.

[6] Mackensen F, Paulus WE, Max R, Ness T (2014). Ocular changes during pregnancy. Dtsch Arztebl Int.

[7] Kubicka-Trzaska A, Karska-Basta I, Kobylarz J, Romanowska-Dixon B (2008). Pregnancy and the eye. Klin Ocz.

[8] Li LJ, Tan KH, Aris IM, Man REK, Gan ATL, Chong YS, Saw SM, Gluckman P, Wong TY, Lamoureux E (2018). Retinal vasculature and 5-year metabolic syndrome among women with gestational diabetes mellitus. Metabolism..

[9] Boone MI, Farber ME, Jovanovic-Peterson L, Peterson CM (1989). Increased retinal vascular tortuosity in gestational diabetes mellitus. Ophthalmology..

[10] Li LJ, Kramer M, Tapp RJ, Man RE, Lek N, Cai S (2017). Gestational diabetes mellitus and retinal microvasculature. BMC Ophthalmol.

[11] Yee KH, Tan KH, Aris IM, Lamoureux EL, Chong YS, Wang JJ, Wong TY, Li LJ (2019). History of gestational diabetes mellitus and postpartum maternal retinal microvascular structure and function. Diabet Med.

[12] Lee J, Rosen R (2016). Optical coherence tomography angiography in diabetes. Curr Diab Rep.

[13] La Spina C, Carnevali A, Marchese A, Querques G, Bandello F (2017). Reproducibility and reliability of optical coherence tomography angiography for foveal avascular zone evaluation and measurement in different settings. Retina..

[14] Shahlaee A, Pefkianaki M, Hsu J, Ho AC (2016). Measurement of foveal avascular zone dimensions and its reliability in healthy eyes using optical coherence tomography angiography. Am J Ophthalmol.

[15] Spaide RF, Fujimoto JG, Waheed NK, Sadda SR, Staurenghi G (2018). Optical coherence tomography angiography. Prog Retin Eye Res.

[16] Kashani AH, Chen CL, Gahm JK, Zheng F, Richter GM, Rosenfeld PJ, Shi Y, Wang RK (2017). Optical coherence tomography angiography: a comprehensive review of current methods and clinical applications. Prog Retin Eye Res.

[17] Ciloglu E, Okcu NT, Dogan NC (2019). Optical coherence tomography angiography findings in preeclampsia. Eye (Lond).

[18] Chanwimol K, Balasubramanian S, Nassisi M, Gaw SL, Janzen C, Sarraf D, Sadda SR, Tsui I (2019). Retinal vascular changes during pregnancy detected with optical coherence tomography angiography. Invest Ophthalmol Vis Sci.

[19] Kiziltunc PB, Varli B, Buyuktepe TC, Atilla H (2020). Ocular vascular changes during pregnancy: an optical coherence tomography angiography study. Graefes Arch Clin Exp Ophthalmol.

[20] Liu G, Xu D, Wang F (2018). New insights into diabetic retinopathy by OCT angiography. Diabetes Res Clin Pract.

[21] Dimitrova G, Chihara E, Takahashi H, Amano H, Okazaki K (2017). Quantitative retinal optical coherence tomography angiography in patients with diabetes without diabetic retinopathy. Invest Ophthalmol Vis Sci.

[22] Al-Sheikh M, Akil H, Pfau M, Sadda SR (2016). Swept-source OCT angiography imaging of the foveal avascular zone and macular capillary network density in diabetic retinopathy. Invest Ophthalmol Vis Sci.

[23] Chong YS, Cai S, Lin H, Soh SE, Lee YS, Leow MK, Chan YH, Chen L, Holbrook JD, Tan KH, Rajadurai VS, Yeo GS, Kramer MS, Saw SM, Gluckman PD, Godfrey KM, Kwek K, GUSTO study group (2014). Ethnic differences translate to inadequacy of high-risk screening for gestational diabetes mellitus in an Asian population: a cohort study. BMC Pregnancy Childbirth.

[24] Alberti KG, Zimmet PZ (1998). Definition, diagnosis and classification of diabetes mellitus and its complications. Part 1: diagnosis and classification of diabetes mellitus provisional report of a WHO consultation. Diabet Med.

[25] Tokayer J, Jia Y, Dhalla AH, Huang D (2013). Blood flow velocity quantification using split-spectrum amplitude-decorrelation angiography with optical coherence tomography. Biomed Opt Express.

[26] Spaide RF, Fujimoto JG, Waheed NK (2015). Image artifacts in optical coherence tomography angiography. Retina..

[27] Lauermann JL, Woetzel AK, Treder M, Alnawaiseh M, Clemens CR, Eter N, Alten F (2018). Prevalences of segmentation errors and motion artifacts in OCT-angiography differ among retinal diseases. Graefes Arch Clin Exp Ophthalmol.

[28] Oian P, Maltau JM (1987). Calculated capillary hydrostatic pressure in normal pregnancy and preeclampsia. Am J Obstet Gynecol.

[29] Cankaya C, Bozkurt M, Ulutas O (2013). Total macular volume and foveal retinal thickness alterations in healthy pregnant women. Semin Ophthalmol.

[30] Ito S, Goto H, Kuniyasu K, Shindo M, Yamada M, Tanaka K, Toh GT, Sawa M, Inagaki M, Bartek J, Masai H (2019). Cdc7 kinase stimulates Aurora B kinase in M-phase. Sci Rep.

[31] Meng L, Wang Y, Zhang L, McDonagh DL (2019). Heterogeneity and variability in pressure autoregulation of organ blood flow: lessons learned over 100+ years. Crit Care Med.

[32] Panerai RB (1998). Assessment of cerebral pressure autoregulation in humans--a review of measurement methods. Physiol Meas.

[33] Bata AM, Fondi K, Witkowska KJ, Werkmeister RM, Hommer A, Vass C, Resch H, Schmidl D, Popa-Cherecheanu A, Chua J, Garhöfer G, Schmetterer L (2019). Optic nerve head blood flow regulation during changes in arterial blood pressure in patients with primary open-angle glaucoma. Acta Ophthalmol.

[34] Kornfield TE, Newman EA (2014). Regulation of blood flow in the retinal trilaminar vascular network. J Neurosci.

[35] Duan A, Bedggood PA, Bui BV, Metha AB (2016). Evidence of flicker-induced functional hyperaemia in the smallest vessels of the human retinal blood supply. PLoS One.

[36] Son T, Wang B, Thapa D, Lu Y, Chen Y, Cao D, Yao X (2016). Optical coherence tomography angiography of stimulus evoked hemodynamic responses in individual retinal layers. Biomed Opt Express.

[37] Zhao D, He Z, Wang L, Fortune B, Lim JKH, Wong VHY, Nguyen CTO, Bui BV (2020). Response of the trilaminar retinal vessel network to intraocular pressure elevation in rat eyes. Invest Ophthalmol Vis Sci.

[38] Augustin M, Fialova S, Fischak C, Schmetterer L, Hitzenberger CK, Baumann B (2017). Ocular fundus pulsations within the posterior rat eye: chorioscleral motion and response to elevated intraocular pressure. Sci Rep.

[39] Herman IM, D'Amore PA (1985). Microvascular pericytes contain muscle and nonmuscle actins. J Cell Biol.

[40] Peppiatt CM, Howarth C, Mobbs P, Attwell D (2006). Bidirectional control of CNS capillary diameter by pericytes. Nature..

[41] Li H, Bui BV, Cull G, Wang F, Wang L (2017). Glial cell contribution to basal vessel diameter and pressure-initiated vascular responses in rat retina. Invest Ophthalmol Vis Sci.

[42] Patel P, Harris A, Toris C, Tobe L, Lang M, Belamkar A, Ng A, Verticchio Vercellin AC, Mathew S, Siesky B (2018). Effects of sex hormones on ocular blood flow and intraocular pressure in primary open-angle glaucoma: a review. J Glaucoma.

[43] Liu G, Keyal K, Wang F (2017). Interocular symmetry of vascular density and association with central macular thickness of healthy adults by optical coherence tomography angiography. Sci Rep.

[44] Dimitrova G, Chihara E (2019). Implication of deep-vascular-layer alteration detected by optical coherence tomography angiography for the pathogenesis of diabetic retinopathy. Ophthalmologica..

[45] Kaizu Y, Nakao S, Arima M, Wada I, Yamaguchi M, Sekiryu H (2019). Capillary dropout is dominant in deep capillary plexus in early diabetic retinopathy in optical coherence tomography angiography. Acta Ophthalmol.

[46] Schock H, Zeleniuch-Jacquotte A, Lundin E, Grankvist K, Lakso HA, Idahl A, Lehtinen M, Surcel HM, Fortner RT (2016). Hormone concentrations throughout uncomplicated pregnancies: a longitudinal study. BMC Pregnancy Childbirth.

[47] Toker E, Yenice O, Akpinar I, Aribal E, Kazokoglu H (2003). The influence of sex hormones on ocular blood flow in women. Acta Ophthalmol Scand.

